# Draft Genome Sequence of Klebsiella pneumoniae UMB8492, Isolated from the Female Urinary Tract

**DOI:** 10.1128/MRA.00405-20

**Published:** 2020-05-14

**Authors:** Suma Gondi, Taylor Miller-Ensminger, Adelina Voukadinova, Alan J. Wolfe, Catherine Putonti

**Affiliations:** a Department of Biology, Loyola University Chicago, Chicago, Illinois, USA; b Bioinformatics Program, Loyola University Chicago, Chicago, Illinois, USA; c Department of Microbiology and Immunology, Stritch School of Medicine, Loyola University Chicago, Maywood, Illinois, USA; d Department of Computer Science, Loyola University Chicago, Chicago, Illinois, USA; University of Arizona

## Abstract

Here, we present the draft genome sequence for Klebsiella pneumoniae strain UMB8492, isolated from a urine sample from a female patient with overactive bladder (OAB) symptoms. The assembled genome has a length of 5,660,575 bp, a G+C content of 57.19%, and an *N*_50_ value of 136,315 bp.

## ANNOUNCEMENT

Klebsiella pneumoniae is a Gram-negative bacterium that is commonly found in the gastrointestinal tract of healthy animals and humans (1, 2). It can also cause urinary tract, respiratory tract, and bloodstream infections (3). K. pneumoniae is a major cause of hospital-acquired infections, and surveys from several different countries estimate that K. pneumoniae accounts for 2 to 6% of hospital-acquired UTIs (3, 4). K. pneumoniae is also classified as a multidrug-resistant (MDR) pathogen, and thus there are limited treatment options (1). Here, we present the draft genome of K. pneumoniae UMB8492, isolated from a catheterized urine sample. While K. pneumoniae is frequently associated with UTIs, the woman from whom it was isolated did not have a UTI but rather had symptoms of overactive bladder (OAB).

K. pneumoniae UMB8492 was isolated from a previously conducted institutional review board (IRB)-approved study (Loyola University Chicago, IRB no. LU207102) using the expanded quantitative urinary culture (EQUC) protocol (5). The genus and species of Klebsiella pneumoniae were confirmed using matrix-assisted laser desorption ionization–time of flight (MALDI-TOF) mass spectrometry before the culture was frozen at −80°C. The EQUC method was also used to isolate strains of Lactobacillus jensenii, Corynebacterium coyleae, Lactobacillus iners, Corynebacterium aurimucosum, and Staphylococcus epidermidis from this same woman’s urinary microbiome. From the freezer stock of UMB8492, an aliquot was streaked onto a Columbia nalidixic acid (CNA) agar plate and incubated at 35°C with 5% CO_2_ for 24 h. A single colony was isolated, and liquid LB medium was inoculated and grown overnight with shaking at 37°C. DNA was isolated from this liquid culture using the Qiagen DNeasy blood and tissue kit following the Gram-positive protocol with the following exceptions. We used 230 μl of lysis buffer (180 μl of 20 mM Tris-Cl, 2 mM sodium EDTA, and 1.2% Triton X-100 and 50 μl of lysozyme) in step 2 and incubated the sample at 56°C for 10 minutes in step 5. The DNA quality was determined using a Qubit fluorometer. DNA was sent to the Microbial Genome Sequencing Center (MiGS) at the University of Pittsburgh for sequencing. MiGS prepared the libraries using the Illumina Nextera kit and sequenced the library on the NextSeq 550 platform. In total, 1,712,978 pairs of 2 × 150-bp reads were generated. These raw reads were trimmed using Sickle v1.33 (https://github.com/najoshi/sickle), using default parameters, and assembled using SPAdes v3.13.0 with the “only-assembler” option for k values of 55, 77, 99, and 127 (6). The genome assembly was annotated using the NCBI Prokaryotic Genome Annotation Pipeline (PGAP) v4.11 (7). The genome coverage was calculated using BBMap v38.47 (https://sourceforge.net/projects/bbmap/) with default parameters.

K. pneumoniae UMB8492 has a draft genome size of 5,660,575 bp, assembled into 127 contigs with an *N*_50_ value of 136,315 bp, and a G+C content of 57.19%. The genome coverage is 77×. PGAP annotation identified 5,508 protein-coding genes and 79 tRNAs. This genome sequence provides an additional genome resource for the study of uropathogens.

### Data availability.

This whole-genome shotgun project has been deposited in GenBank under accession no. JAAUVU000000000. The version described in this paper is the first version, JAAUVU010000000. The raw sequencing reads have been deposited in the SRA under accession no. SRR11441023.
